# Vegan Diet and Food Costs Among Adults With Overweight: A Secondary Analysis of a Randomized Clinical Trial

**DOI:** 10.1001/jamanetworkopen.2023.32106

**Published:** 2023-09-05

**Authors:** Hana Kahleova, Macy Sutton, Cristina Maracine, Daniel Nichols, Pablo Monsivais, Richard Holubkov, Neal D. Barnard

**Affiliations:** 1 Physicians Committee for Responsible Medicine, Washington, DC; 2 George Washington School of Public Health, Washington, DC; 3 SUNY Upstate Medical University, Syracuse, New York; 4 Department of Nutrition and Exercise Physiology, Elson S. Floyd College of Medicine, Washington State University, Spokane; 5 School of Medicine, University of Utah, Salt Lake City; 6 George Washington University School of Medicine and Health Sciences, Washington, DC

## Abstract

This secondary analysis of a randomized clinical trial investigates the effects of a vegan diet on total food costs per day.

## Introduction

Vegan diets are recognized for numerous health benefits. However, given that food costs may represent a barrier to dietary change, the costs of vegan diets merit examination.

As reported previously in a randomized clinical trial (RCT),^1^ an ad libitum low-fat vegan diet resulted in weight loss, improved body composition, and increased insulin sensitivity in overweight adults. This secondary analysis of that RCT assessed food costs. As the prices of staple foods, such as rice and beans, are much lower compared with meat and dairy, it was hypothesized that food costs would be reduced on a vegan diet.

## Methods

The methods have been previously described.^1^ In brief, this randomized, open parallel study was conducted between January 2017 and February 2019 in Washington, DC (trial protocol in Supplement 1). This study followed the CONSORT reporting guideline. The protocol was approved by the Chesapeake institutional review board. All participants provided written informed consent.

Participants were randomly assigned to a vegan or control group in a 1:1 ratio (eFigure in Supplement 2). The vegan group was asked to follow an ad libitum low-fat vegan diet consisting of fruits, vegetables, grains, and legumes, while the control group was requested to make no diet changes. Energy intake and food costs were not limited for either group. At baseline and week 16, a 3-day dietary record (2 weekdays and 1 weekend day) was completed by each participant and analyzed by a registered dietitian certified in the Nutrition Data System for Research.^2^

For the food cost assessment, intakes from dietary records were linked to a database of national food prices,^3^ using the US Department of Agriculture Thrifty Food Plan.^4^ Two independent reviewers (C.M. and D.N.), blinded to group assignment, linked it with food groups from the dietary analysis software.^2^ Linking accuracy was verified by a senior researcher (P.M.), also blinded to group assignment. A repeated measure analysis of variance was used by a statistician blinded to dietary interventions. All significance reporting is 2-sided with type I error of .05. Two-sample *t*-tests were used to assess the differences between treatment groups, and 1-sample *t*-tests to assess significance of changes within each group. Statistical analysis was conducted using SAS version 9.4 (SAS Institute) in May 2023.

## Results

Of 3115 people screened by telephone, 244 adults with overweight met participation criteria and were randomly assigned to the vegan (n = 122; 105 [86.1%] female; 60 [49.2%] Black; 57 [46.7%] White; mean [SD] age, 52.9 [10.3] years) or control (n = 122; 106 [86.9%] female; 53 [43.4%] Black; 60 [49.2%] White; mean [SD] age, 56.7 [12.8] years) groups. The analysis included 223 (91.0%) participants who completed all aspects of the study, including the final diet records.

Mean (95% CI) total food costs per day decreased in the vegan group by approximately 16%, compared with no significant change in the control group. The difference between the groups was significant (Figure, Table). The biggest savings were on meat and dairy. These savings outweighed the increased spending on vegetables, fruits, legumes, whole grains, meat alternatives, and dairy alternatives.

**Figure.**
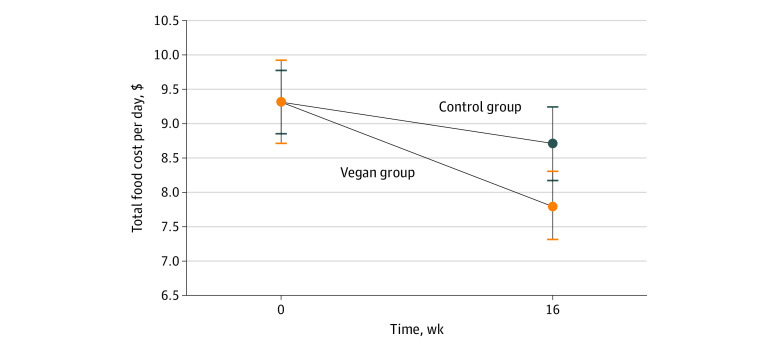
Changes in Total Food Costs in the Vegan and Control Group From Baseline to Week 16 The data are shown as means with 95% CIs.

**Table.** Changes in Economic Costs From Specific Food Groups at Baseline and Week 16 in the Control and Vegan Groups

Food groups	Economic costs (95% CI), $/d					*P* value
	Control group		Vegan group		Treatment effect size	
	Baseline	Week 16	Baseline	Week 16		
Total economic costs	9.31 (8.71 to 9.92)	8.71 (8.17 to 9.24)	9.31 (8.85 to 9.77)	7.80 (7.31 to 8.30)	−0.90 (−1.73 to −0.07)	.03
Fruits	0.89 (0.76 to 1.03)	0.96 (0.74 to 1.17)	0.95 (0.80 to 1.10)	1.42 (1.25 to 1.58)	0.40 (0.11 to 0.68)	.006
Vegetables	2.08 (1.85 to 2.31)	2.01 (1.81 to 2.21)	2.03 (1.83 to 2.23)	3.00 (2.72 to 3.28)	1.03 (0.64 to 1.42)	<.001
Legumes	0.16 (0.12 to 0.21)	0.14 (0.10 to 0.18)	0.16 (0.12 to 0.20)	0.43 (0.35 to 0.52)	0.30 (0.20 to 0.40)	<.001
Grains total	1.43 (1.27 to 1.58)	1.07 (0.94 to 1.20)	1.55 (1.39 to 1.70)	1.28 (1.15 to 1.40)	0.08 (−0.17 to 0.34)	.52
Whole grains	0.44 (0.37 to 0.52)	0.33 (0.27 to 0.40)	0.42 (0.35 to 0.49)	0.62 (0.55 to 0.69)	0.30 (0.17 to 0.44)	<.001
Some whole grains	0.05 (0.02 to 0.08)	0.08 (0.05 to 0.11)	0.06 (0.03 to 0.09)	0.09 (0.06 to 0.12)	−0.002 (−0.06 to 0.05)	.94
Refined grains	0.93 (0.79 to 1.08)	0.66 (0.54 to 0.77)	1.06 (0.94 to 1.19)	0.57 (0.47 to 0.66)	−0.22 (−0.44 to 0.003)	.050
Total meat	1.85 (1.56 to 2.14)	1.87 (1.55 to 2.18)	1.79 (1.54 to 2.04)	0.03 (0.00 to 0.07)	−1.77 (−2.19 to −1.35)	<.001
Red meat	0.39 (0.27 to 0.51)	0.36 (0.24 to 0.47)	0.27 (0.19 to 0.34)	0.00 (0.00 to 0.00)	−0.23 (−0.37 to −0.09)	.002
White meat	1.22 (0.98 to 1.46)	1.19 (0.92 to 1.47)	1.29 (1.06 to 1.51)	0.03 (0.01 to 0.06)	−1.23 (−1.63 to −0.84)	<.001
Processed meat	0.22 (0.15 to 0.29)	0.29 (0.18 to 0.41)	0.21 (0.15 to 0.26)	0.002 (0.00 to 0.01)	−0.28 (−0.41 to −0.14)	<.001
Fried meat	0.03 (0.004 to 0.05)	0.03 (0.01 to 0.05)	0.03 (0.01 to 0.06)	0.00 (0.00 to 0.00)	−0.03 (−0.07 to 0.00)	.056
Meat alternatives	0.18 (0.08 to 0.28)	0.17 (0.07 to 0.28)	0.16 (0.09 to 0.24)	0.59 (0.46 to 0.73)	0.44 (0.25 to 0.62)	<.001
Eggs	0.12 (0.08 to 0.16)	0.1 (0.07 to 0.13)	0.14 (0.11 to 0.17)	0.0 (0.0 to 0.001)	−0.13 (−0.18 to −0.08)	<.001
Nuts and seeds total	0.30 (0.22 to 0.38)	0.26 (0.2 to 0.32)	0.26 (0.18 to 0.34)	0.07 (0.05 to 0.09)	−0.15 (−0.27 to −0.03)	.015
Daiy total	0.85 (0.71 to 0.99)	0.77 (0.62 to 0.92)	0.88 (0.74 to 1.01)	0.05 (0.02 to 0.08)	−0.74 (−0.96 to −0.52)	<.001
Full-fat	0.50 (0.40 to 0.61)	0.38 (0.28 to 0.48)	0.49 (0.38 to 0.59)	0.01 (0.00 to 0.01)	−0.36 (−0.52 to −0.20)	<.001
Low-fat	0.21 (0.15 to 0.27)	0.18 (0.13 to 0.23)	0.24 (0.18 to 0.30)	0.003 (0.00 to 0.01)	−0.21 (−0.30 to −0.11)	<.001
No-fat	0.11 (0.07 to 0.15)	0.14 (0.08 to 0.19)	0.12 (0.07 to 0.16)	0.00 (0.00 to 0.00)	−0.14 (−0.22 to −0.07)	<.001
Dairy alternatives	0.13 (0.09 to 0.18)	0.1 (0.06 to 0.15)	0.18 (0.13 to 0.24)	0.32 (0.26 to 0.39)	0.17 (0.07 to 0.26)	<.001
Added fats	0.26 (0.22 to 0.30)	0.26 (0.22 to 0.30)	0.23 (0.20 to 0.26)	0.10 (0.08 to 0.12)	−0.14 (−0.20 to −0.08)	<.001
Animal fats	0.05 (0.03 to 0.07)	0.05 (0.03 to 0.08)	0.04 (0.03 to 0.06)	0.00 (0.00 to 0.00)	−0.04 (−0.07 to −0.02)	.003
Plant oils	0.21 (0.18 to 0.24)	0.21 (0.18 to 0.24)	0.19 (0.17 to 0.22)	0.10 (0.07 to 0.12)	−0.10 (−0.15 to −0.05)	<.001
Sweets and desserts	0.26 (0.21 to 0.30)	0.28 (0.22 to 0.33)	0.26 (0.21 to 0.31)	0.31 (0.23 to 0.39)	0.03 (−0.09 to 0.14)	.66
Sweetened beverages	0.23 (0.16 to 0.31)	0.21 (0.13 to 0.29)	0.14 (0.10 to 0.19)	0.06 (0.03 to 0.08)	−0.07 (−0.16 to 0.02)	.15

## Discussion

This secondary analysis of an RCT found that a low-fat vegan diet was associated with an approximately 16% decrease in total food costs. In addition to health benefits, a vegan diet may have economic advantages. A 2021 study estimated that diets including less animal and more plant foods were up to 25% to 29% less expensive than omnivorous diets.^5^ A large US Internet survey found that food expenditures for vegetarians were lower than for their meat-eating counterparts.^6^

The strengths of the current study include a randomized, parallel design, which accounted for seasonal effects. The study also has limitations. Food costs were based on self-reported diet records and did not include supplements or medications. Food cost estimates in the Thrifty Food Plan are conservative and exclude alcohol. The participants were research volunteers and may not represent the general population.

## Notes

Supplement 1.
Trial Protocol

Supplement 2. **eFigure.** Participant Flow Chart

Supplement 3.
Data Sharing Statement
