# Supplementary material for: Vegan Diet and Food Costs Among Adults With Overweight: A Secondary Analysis of a Randomized Clinical Trial
Source: JAMA Netw Open. 2023 Sep 5;6(9):e2332106. doi: 10.1001/jamanetworkopen.2023.32106 (PMC10481244; doi:10.1001/jamanetworkopen.2023.32106)
Supplement: Supplement 2. — eFigure. Participant Flow Chart [file jamanetwopen-e2332106-s002.pdf]

Supplemental Online Content

Kahleova H, Sutton M, Maracine C, et al. Vegan diet and food costs among adults with overweight: a secondary analysis of a randomized clinical trial. *JAMA Netw Open.* 2023;6(9):e2332106. doi:10.1001/jamanetworkopen.2023.32106

eFigure. Participant Flow Chart

This supplemental material has been provided by the authors to give readers additional information about their work.

© 2023 Kahleova H et al. *JAMA Network Open.*

eFigure. Participant Flow Chart.

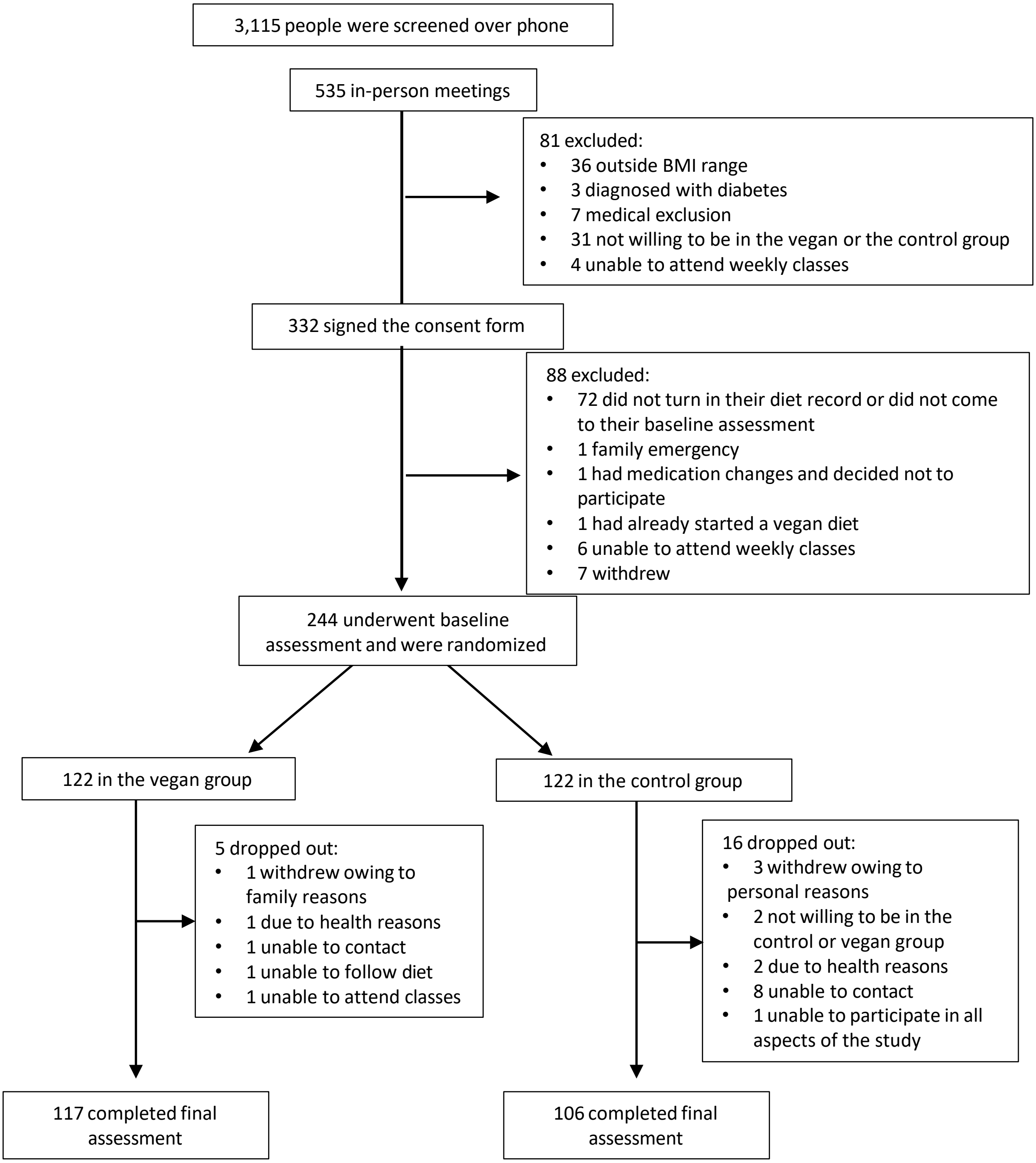

© 2023 Kahleova H et al. *JAMA Network Open.*
