# Supplementary material for: Vegan Diet and Food Costs Among Adults With Overweight: A Secondary Analysis of a Randomized Clinical Trial
Source: JAMA Netw Open. 2023 Sep 5;6(9):e2332106. doi: 10.1001/jamanetworkopen.2023.32106 (PMC10481244; doi:10.1001/jamanetworkopen.2023.32106)
Supplement: Supplement 3. — Data Sharing Statement [file jamanetwopen-e2332106-s003.pdf]

Data Sharing Statement

Kahleova. Vegan Diet and Food Costs Among Adults With Overweight. *JAMA Netw Open*. Published September 05, 2023. doi:10.1001/jamanetworkopen.2023.32106

Data
Data available: Yes
Data types: Deidentified participant data
How to access data: Deidentified participant data will be available upon request at hana.kahleova@gmail.com.
When available: With publication

Supporting Documents
Document types: None

Additional Information
Who can access the data: The data will be made available to researchers who received permission from their institution.
Types of analyses: systematic reviews and meta-analyses
Mechanisms of data availability: With a signed data agreement.
